# The importance of the microbiome and metabolome in health and disease of dogs and cats

**DOI:** 10.1186/1751-0147-57-S1-K6

**Published:** 2015-09-25

**Authors:** Jan Suchodolski

**Affiliations:** 1 GI Lab, Texas A&M University, College Station, TX, USA

## 

The canine intestinal tract harbors a highly complex microbial ecosystem. Various studies have reported changes in microbial communities in acute and chronic gastrointestinal diseases, especially in inflammatory bowel disease (IBD) and acute diarrhea. Most commonly observed are decreases in the bacterial phyla Firmicutes (i.e., Lachnospiraceae, Ruminococcaceae, Faecalibacterium) and Bacteroidetes, with concurrent increases in Proteobacteria (i.e. *Escherichia coli*). Due to the importance of microbial and host derived metabolites for intestinal health, it is important to better understand the functional consequences of microbial dysbiosis. Novel approaches such as shotgun sequencing of DNA allow describing the functional changes in the bacterial metagenome in gastrointestinal disease. Furthermore, wide scale and untargeted measurements of metabolic products derived by the host and the microbiota in intestinal samples allow a better understanding of the functional alterations that occur in gastrointestinal disease. For example, changes in bile acid metabolism, short-chain fatty acid concentrations, and tryptophan pathways have recently been reported in humans and dogs. Also, metabolites associated with the pentose phosphate pathway were reported to be differentially expressed in gastrointestinal inflammation and indicate the presence of oxidative stress in dogs with IBD. Of interest is that these changes did not correlate with clinical activity, suggesting the presence of ongoing inflammation. Better understanding of the interactions of microbial-derived metabolites and the host will yield insights into the pathophysiology of gastrointestinal diseases and may be a new avenue for therapeutic approaches. Furthermore, some of these metabolic pathways can be used for monitoring of therapy success.

